# Characterization and oncolytic virus targeting of FAP-expressing tumor-associated pericytes in glioblastoma

**DOI:** 10.1186/s40478-020-01096-0

**Published:** 2020-12-11

**Authors:** Ming Li, Guoping Li, Juri Kiyokawa, Zain Tirmizi, Leland G. Richardson, Jianfang Ning, Saumya Das, Robert L. Martuza, Anat Stemmer-Rachamimov, Samuel D. Rabkin, Hiroaki Wakimoto

**Affiliations:** 1 Department of Neurosurgery, Massachusetts General Hospital, Harvard Medical School, 185 Cambridge Street, Boston, MA 02114 USA; 2 Cardiovascular Research Center, Massachusetts General Hospital, Harvard Medical School, Boston, MA 02114 USA; 3 Department of Pathology, Massachusetts General Hospital, Harvard Medical School, Boston, MA 02114 USA; 4 Present Address: Department of Neurosurgery, University of Minnesota Medical School, Minneapolis, MN 55455 USA

**Keywords:** Glioblastoma, Tumor-associated fibroblasts, Pericytes, Oncolytic virus, FAP

## Abstract

Cancer-associated fibroblasts (CAFs) are activated fibroblasts constituting the major stromal components in many types of cancer. CAFs contribute to hallmarks of cancer such as proliferation, invasion and immunosuppressive tumor microenvironment, and are associated with poor prognosis of patients with cancer. However, in glioblastoma (GBM), the most common and aggressive primary malignant brain tumor, our knowledge about CAFs or CAF-like stromal cells is limited. Here, using commonly accepted CAF markers, we characterized CAF-like cell populations in clinical glioma specimens and datasets along with mouse models of GBM. We found that tumor-associated pericytes marked by co-expression of fibroblast activation protein α (FAP) and PDGFRβ represent major stromal cell subsets in both human GBM and mouse GBM models, while a fraction of mesenchymal neoplastic cells also express FAP in patient tumors. Since oncolytic viruses can kill cancer cells and simultaneously modulate the tumor microenvironment by impacting non-neoplastic populations such as immune cells and tumor vasculature, we further investigated the ability of oncolytic viruses to target GBM-associated stromal cells. An oncolytic adenovirus, ICOVIR15, carrying ∆24-E1A and an RGD-fiber, infects and depletes FAP+ pericytes as well as GBM cells in murine GBM. Our study thus identifies FAP+/PDGFRβ+ pericytes as a major CAF-like stromal cell population in GBM, and highlights the unique property of this oncolytic adenovirus to target both GBM cells and GBM-associated stromal FAP+ cells.

## Introduction

Cancer-associated fibroblasts (CAFs) are activated fibroblasts constituting the major stromal component in many types of cancer, including breast, lung, and pancreatic cancers [1–3]. A growing body of evidence indicates that CAFs play a crucial role in tumor development and progression [4]. CAFs drive the synthesis and remodeling of extracellular matrix, closely interact with cancer cells to promote their proliferation and migration, and participate in angiogenesis and inflammation via the secretion of cytokines. Recent research further reveals that CAFs contribute to cancer progression via evasion of immune surveillance, and providing resistance to immunotherapy [5–7]. CAFs are heterogenous populations that display distinct protein profiles and have multiple cells of origin, including resident fibroblasts, bone marrow-derived mesenchymal stem cells, and cancer stem cells, prompting research aimed at understanding the specific biological functions of CAF subsets. Although there is no specific single marker to universally define CAFs, several proteins have been shown to help identify CAFs, including fibroblast activation protein α (FAP), α-smooth muscle actin (αSMA), fibroblast specific protein 1 (FSP1), and platelet-derived growth factor receptors (PDGFR) α and β [8, 9]. Among these, FAP, a trans-membrane cell surface protein with serine peptidase activity, is one of the most commonly used and reliable CAF markers due to its selective expression in activated or cancer-educated fibroblasts, and functions to suppress anti-tumor immune cells, promote tumor growth, and drive epithelial–mesenchymal transition [9–11]. Clinically, the abundance of FAP+ CAFs is associated with poor prognosis of patients in several types of cancer [12–14]. Thus, CAFs, particularly those marked by FAP, are considered a promising therapeutic target for cancer therapy.

Compared with solid cancers outside the CNS, our knowledge about CAFs or CAF-like stromal cells present in glioblastoma (GBM), the most common and aggressive primary neuroepithelial tumor in the brain, is limited [15, 16]. Analogous to other stroma-rich malignancies, GBM has a complex tumor microenvironment that is characterized by a mix of neoplastic and non-neoplastic cell types including immune, neuronal and vascular cells, and extracellular matrix components. Crosstalk between heterogenous cell types shapes the immune-suppressive tumor microenvironment, a hallmark of GBM, rendering immunotherapy less effective. It has been reported that stromal cells with a mesenchymal phenotype expressing CAF markers are present in GBM [17–19]. Experimentally, co-implantation of GBM-associated stromal cells promoted tumor growth and angiogenesis in a human GBM cell line U87-based model [18, 20]. Radioisotope-labeled FAP-binding ligands showed accumulation in PET imaging of patients with malignant glioma, suggesting a diagnostic utility of FAP [19]. However, both the identity and role of FAP+ stromal or CAF-like cells in the GBM tumor microenvironment are poorly understood. Moreover, therapeutic targeting of GBM-associated CAF-like cells has not been explored.

In the current work, we characterize CAF-like cell populations in clinical glioma specimens and datasets along with mouse models of GBM. We found that pericytes marked by co-expression of FAP and PDGFRβ represent the major stromal components shared by GBM patients and mouse models. Because of their ability to selectively kill tumor cells without hurting normal tissue, oncolytic viruses are a promising modality in the treatment of cancer including GBM, and can simultaneously modulate the tumor microenvironment by impacting non-neoplastic populations such as immune cells and tumor vasculature [21–23]. We demonstrate that an oncolytic adenovirus can target GBM-associated FAP+ stromal pericytes, in addition to killing tumor cells.

## Materials and methods

### Cells

Mouse 005 GBM stem-like cells (GFP positive) were provided by Dr. I Verma (Salk Institute) and have been described [21, 24]. They were cultured as spheres in EF20 medium composed of Neurobasal medium (Thermo Fisher Gibco) supplemented with 3 mM l-Glutamine (Corning Mediatech), 1 × B27 supplement (Thermo Flasher Gibco), 0.5 × N2 supplement (Thermo Fisher Gibco), 2 μg/ml heparin (Sigma, St Louis, MO), 20 ng/ml recombinant human epidermal growth factor (R&D Systems, Minneapolis, MN), 20 ng/ml recombinant human fibroblast growth factor-2 (PeproTech, Rocky Hill, NJ), and 0.5 × penicillin G/streptomycin sulfate/amphotericin B complex (Corning Mediatech) at 37 °C and 5% CO_2_. To passage cells, neurospheres were dissociated with the Neurocult chemical dissociation kit (Stem Cell Technologies). Mouse GBM GL261 cells were obtained from the National Cancer Institute and grown in Dulbecco’s Modified Eagle Medium (DMEM) supplemented with 10% fetal calf serum (FCS). Cells were confirmed to be mycoplasma free (LookOut Mycoplasma kit, Sigma) and used at low passage number.

### Mouse GBM tissue harvest

Mouse 005 GBM tumor tissues were excised, dissected and cut into 1 mm fragments in DMEM. Tissue fragments were transferred to a conical tube, spun at 1100 rpm, and digested with Accutase and DNase I (10 U/ml; Promega) at 37 °C for 10 min. Tissue was triturated, and passed through a 40-μm cell strainer to yield a single cell suspension. These cells were used for downstream experiments after centrifuge at 1500 rpm and supernatant aspiration.

### Flow cytometry sorting

Cells were adjusted to a concentration of 1 × 10^6^ cells/mL in 100 μl cold PBS, washed with FACS buffer (2% PBS and 0.5 mM EDTA in PBS), and blocked with FcR blocker (Miltenyi) for 20 min at 4 °C, followed by incubation with anti-FAP primary antibody (1:100; Additional file 1: Table S1) for 60 min in dark at 4 C. After 3 washes, cells were incubated with APC-anti-Rabbit IgG (R and D Systems, 1:100) on ice in the dark for 40 min. After 2 washes with FACS buffer, cells were transferred to FACS tubes and subject to cell sorting using a FACS machine (BD). Cells were collected in DMEM, immediately centrifuged at 1500 rpm for 5 min, and the pellets stored at − 80 °C freezer for later RNA extract.

### Immunofluorescence staining of cells

Tissue-derived 005 GBM cells (1 × 10^5^) were plated on round glass coverslips in 24-well plates in DMEM with 10% FCS, and treated with mock or ICOVIR15 (MOI = 10). On day 2, 4 and 7 post-infection, cells were fixed with 4% paraformaldehyde for 10 min, washed with PBS, blocked by 5% bovine serum albumin (BSA), and incubated with primary antibody to FAP and hexon (Additional file 1: Table S1) overnight at 4 °C in a humidified chamber. Next day, cells were washed with PBS, incubated with secondary antibodies AMCA-anti-goat IgG (1:250; Jackson ImmunoResearch) and Cy3 anti-rabbit IgG (1:250; Jackson ImmunoResearch) for 1 h at room temperature, and slides were mounted with VectaShield (DAPI included, Vector Laboratories). Staining was imaged with a Nikon 90i microscope and quantified at three or more randomly chosen high power fields per coverslip.

### Immunofluorescence or immunohistochemistry (IHC) staining on formalin-fixed paraffin-embedded tissue sections

Each patient tumor was assigned to MGG(number), and some tumors have been described previously [25]. Formalin-fixed paraffin-embedded (FFPE) tissue sections were de-paraffinized at 55 °C heating and in xylene, and hydrated with series of graded ethanol (100%, 90–95%, 70%) for 5 min each. After PBS wash, slides were treated by microwave in 10 mM Na Citrate buffer for 15 min for antigen retrieval. After cooling, slides were placed in PBS for 5 min, incubated with 5% BSA for 1 h, and incubated with primary antibodies overnight at 4 °C. Next day, sides were washed with PBS for 5 min three times, and secondary antibodies (Alexa Fluor 546-conjugated anti-mouse IgG and Alexa Fluor 488-conjugated anti-rabbit IgG, Thermo Fisher) were applied for 1 h incubation at room temperature. After 3 PBS washes 5 min each, slides were mounted with anti-fade DAPI solution and cover-glass. Staining was imaged with a Nikon 90i microscope and quantified using at least three high power fields.

For FAP IHC, microwave-treated brain sections were incubated with 3% H_2_O_2_ for 5 min to block endogenous peroxidase and blocked with 5% BSA. Slides were then incubated with anti-FAP primary antibody overnight at 4 °C, washed and incubated with ImmPRESS polymer reagents anti-rabbit (Vector). After washes, brown color was developed with DAB (Dako or Vector). Slides were counter-stained with hematoxylin, washed in running tap water for 5 min, dehydrated with a series of graded ethanol and cleaned with xylene, and mounted in Cytoseal XYL.

Double IHC was performed as previously described [23, 26]. Briefly, brain sections were incubated sequentially with primary antibody, then secondary antibody (HRP-conjugated anti-rabbit Ig, Vector), followed by the development of red or brown color using ImmPACT Vector Red or Brown horseradish peroxidase Substrate Kit (Vector). Next, the same sections were incubated with second primary antibody, then secondary antibody (AP-conjugated anti-rabbit or anti-mouse Ig, Vector), followed by the development of blue color using Vector Blue Alkaline Phosphatase Substrate Kit (Vector). Slides were rinsed with distilled water, air dried, cleaned with xylene, and mounted in Cytoseal XYL. Staining was counted from at least three random fields/tumor section by investigators blinded to the treatment. All primary antibodies used in this work were listed in Additional file 1: Table S1.

### Animal study

C57BL/6 mice (8-week old, females, from Charles River) were anesthetized and fixed in a stereotactic head frame. After midline skin incision, skull was exposed and a burr hole was drilled on the coronal suture at 2.3 mm lateral (right) from the midline (Bregma). Using a Hamilton syringe, 1 × 10^5^ 005 cells in 3 µl PBS were slowly (30 s) injected into the brain at 2.5 mm depth from the brain surface. Three minutes later, the injection needle was withdrawn, the burr hole closed with bone wax, and the wound sutured. On days 18 and 21, 3 μl of PBS, ICOVIR15 [27] (provided by Dr. Ramon Alemany, 1.2 × 10^7^ PFU/mouse), or G47Δ [28] (5X10^5^ PFU/mouse) were injected into the tumor site using the previous burr hole and depth. On day 25, all mice were killed for brain harvest. For the GL261 model, 1 × 10^5^ cells were injected at the same location. On day 20, mice were euthanized for brain removal. All in vivo procedures were approved by the Institutional Animal Care and Use Committee (IACUC) at Massachusetts General Hospital. The viruses used in this study were purified according to prior publications [29, 30].

### Quantitative RT-PCR

Total RNA of sorted cells was extracted with Trizol (Invitrogen) according to the manufacturer’s protocol. First strand cDNA was synthesized using the High-Capacity cDNA Reverse Transcription Kit (Applied Biosystems). Quantitative PCR was performed with SYBR green PCR master mix (Applied Biosystems) in a real-time PCR machine (Step One Plus Real-Time PCR System, Applied Biosystems). β-actin (*Actb*) was used as the house-keeping gene control and the 2^−ΔΔCT^ method was used for determining relative RNA levels.

### Clinical data analysis

FAP RNA levels in GBM and normal brain were analyzed at the UCSC Xena website (https://xena.ucsc.edu/). FAP RNA levels in different grade and IDH status of adult glioma, overall survival of patients with gliomas and relationship of two genes were analyzed using the TCGA and CGGA datasets according to the instruction of GlioVis (https://gliovis.shinyapps.io/GlioVis/). Single cell RNA sequencing data of human GBM was performed at the Gephart lab website (http://www.gbmseq.org/) [31]. Single cell RNA sequencing raw data were downloaded from GSE84465. Data analyses were performed using scRNAseq package R version 4.0.3. Pearson correlation was analyzed by ggpubr package in R. The labeling of different cell types and FAP +/PDGFRB+ cells was done by using cellassign package in R.

### Statistical analysis

Experimental results were analyzed using unpaired two-sided Student’s t test, as indicated in Figure legends (Prism; GraphPad). *p* < 0.05 was considered statistically significant. Statistical analysis of clinical data was done according to the corresponding websites.

## Results

### Increased presence of FAP-positive cells in human malignant gliomas

**Fig. 1:**
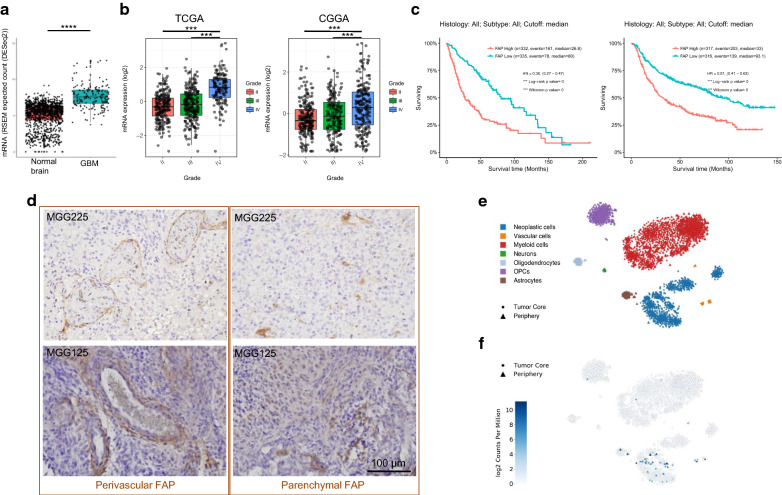
Increased presence of FAP-positive cells in human malignant gliomas. **a** FAP RNA levels (RNAseq) in GBM and normal brain. Analysis of TCGA GBM data (N = 166) and GTEx normal brain tissue data (N = 1141) using UCSC Xena. ****p < 0.0001 (*t* test). **b** FAP RNA levels in different grades of adult glioma. Analysis of the TCGA and CGGA datasets at GlioVis. N = 515 for lower-grade gliomas and N = 152 for grade IV (GBM) in TCGA. ***p < 0.001 (pairwise comparisons of Tukey’s Honest Significant Difference). **c** Kaplan–Meier analysis of overall survival of patients with gliomas using the TCGA and CGGA datasets. **d** Representative images of FAP immunohistochemistry (IHC) in GBM, showing two different staining patterns, perivascular/circular (left) and scattered/parenchymal (right). See Additional file 1: S1 for additional data on FAP IHC. **e**, **f** 2D-tSNE presentation of cell cluster mapping based on single cell RNAseq data of GBM (36 and http://gbmseq.org/) (**e**), and mapping of FAP mRNA on to the same tSNE map (**f**). Cells with differing phenotypes are color-coded in **e**

We first examined the expression of FAP and its potential role as a biomarker in human glioma using two large glioma datasets, TCGA and CGGA. mRNA levels of FAP were significantly elevated in GBM, compared with normal brain (Fig. 1a) and lower grade gliomas (WHO grade II and III, Fig. 1b). FAP transcripts were significantly higher in IDH-wildtype gliomas compared to IDH-mutant counterparts (Additional file 1: Fig. S1a). Survival analysis in the large TCGA and CGGA datasets consistently showed that across gliomas of all malignancy grades, high-level tumor FAP mRNA was associated with poor prognosis with high statistical significance (Fig. 1c).

We next assessed FAP protein expression using immunohistochemistry (IHC) on FFPE specimens of malignant gliomas (13 IDH-wt GBM, 1 IDH-mutant anaplastic astrocytoma and 1 IDH-mutant GBM) collected at MGH. All 15 tumors tested contained cells immuno-positive for FAP to varying degrees (Fig. 1d, Table 1). We noted that the patterns of FAP staining could be classified into two distinct morphological types. One type, more frequent (14/15 cases), consisted of cells clustered in small aggregates within the tumor (parenchymal pattern). The second type consisted of elongated cells wrapped around small vessels (perivascular pattern) and was less frequent (4/15 cases) (Fig. 1d). In some tumors (3/15 cases), both patterns were present (Table 1). To understand the phenotype of FAP+ cells in GBM, we utilized single cell RNA sequencing data derived from 4 GBMs that contain neoplastic as well as a variety of non-neoplastic cell types [31]. Mapping of *FAP* onto this RNA-based single cell atlas showed that the existence of *FAP*-positive cells was mostly limited to: (1) large clusters of neoplastic cells and (2) much smaller clusters of vascular cells (Fig. 1e, f). Thus, human GBM contains cells expressing FAP, which exhibit neoplastic or vascular phenotypes.

**Table 1:** Summary of basic clinical and FAP IHC information of the cohort of 15 malignant gliomas

Sample Number	Pathology	Primary vsersu recurrent	IDH1 Status	Perivascular FAP	Parenchymal FAP
MGG7	GBM	Primary	Wild type	–	++
MGG8	GBM	Primary	Wild type	–	++
MGG63	GBM	Recurrent	Wild type	–	+
MGG65	GBM	Primary	Wild type	–	++
MGG66	GBM	Primary	Wild type	–	+
MGG67	GBM	Primary	Wild type	–	+
MGG81	GBM	Primary	Mutant	–	+
MGG90	GBM	Primary	Wild type	–	++
MGG100	GBM	Primary	Wild type	–	+
MGG125	GBM	Primary	Wild type	+	+
MGG153	GBM	Primary	Wild type	+	+
MGG162	AA	Primary	Mutant	–	+
MGG168	GBM	Recurrent	Wild type	+	–
MGG169	GBM	Primary	Wild type	–	+
MGG225	GBM	Primary	Wild type	+	+

GBM, glioblastoma; AA, anaplastic astrocytoma; −, negative, +, 2% or less; ++, 2–10%

### Tumor-associated pericytes represent the major cell type that expresses FAP in GBM

**Fig. 2:**
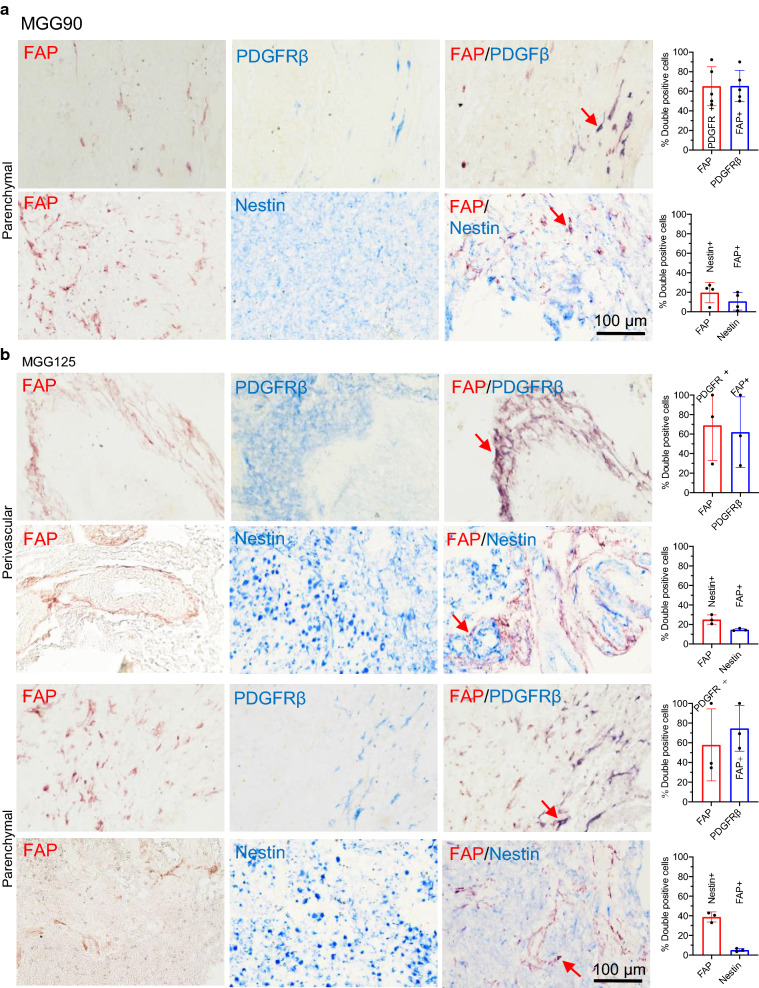
Tumor-associated pericytes represent the major cell type that expresses FAP in GBM. **a**, **b** Double immunohistochemistry (IHC) of FAP (red)/PDGFR (blue), and FAP (red)/nestin (blue) in MGG90 (**a**) and MGG125 GBM (**b**). Left, Representative IHC images of parenchymal and perivascular areas. Right, Quantification of the fraction of double positive cells. Arrows point to representative double positive cells (dark purple)

To better define the phenotypic characteristics of FAP+ cells in human glioma samples, we first used FAP/nestin and FAP/PDGFRβ double immunostaining on human glioma specimens. Nestin and PDGFRβ were chosen as markers for primarily labeling GBM or stem/progenitors and CAFs or pericytes, respectively [9, 32, 33]. In a human GBM (MGG90) that prominently displayed parenchymal FAP with no noticeable perivascular FAP, the majority (~ 60%) of FAP+ cells were PDGFRβ+ and the majority (~ 60%) of PDGFRβ+ cells were FAP+ (Fig. 2a). A minority (~ 15%) of FAP+ cells were nestin+, and only 10% of nestin+ cells were FAP+ (Fig. 2a). Co-staining of FAP and nestin was also present in another GBM (MGG7), indicating that a minor subset of GBM cells express FAP (Additional file 1: Fig S2a). FAP/PDGFRβ-double positive cells were typically elongated, morphologically consistent with pericytes (Fig. 2a). In GBM MGG125, containing both perivascular and parenchymal scattered FAP+ structures-cells, perivascular FAP+ cells were predominantly PDGFRβ+ (~ 70%) and to a much lesser extent nestin+ (20%) (Fig. 2b). As observed in MGG90, scattered parenchymal FAP+ cells were also mostly PDGFRβ+ (~ 60%), with a smaller but substantive fraction (~ 40%) being nestin+, although only about 5% of nestin+ were FAP+ (Fig. 2b). Regardless of the staining patterns, i.e., parenchymal or perivascular, 60–75% of PDGFRβ+ cells were FAP+. On the other hand, the fraction of nestin+ cells that were FAP+ was very small (< 15%) (Fig. 2a, b). Further characterization using double immunofluorescence of FAP with GFAP and Ki67, an astrocyte and proliferation marker, respectively, showed no co-localization of FAP and GFAP and a low proliferative activity of FAP+ cells (Additional file 1: Fig S2b, c). Despite frequent co-expression of FAP and PDGFRβ, there was no co-labeling of FAP with αSMA, a marker commonly used for CAFs, vascular smooth muscle cells and pericytes (Additional file 1: Fig S2d). In IDH-mutant GBM MGG81, co-staining of FAP and mutant IDH1R132H revealed half of FAP+ cells were neoplastic cells (Additional file 1: Fig S3a), and about 70% of FAP+ cells were co-labeled with nestin or PDGFRβ (Additional file 1: Fig. S3b, c). Since CAFs promote TGFβ signaling [34, 35] and TGFβ contributes to immunosuppression in GBM [36] and the maintenance of GBM stem cells [37, 38], we used double staining of FAP and TGF-β1, and found that a fraction of FAP+ cells (~ 17%) were co-labeled with TGF-β1 in GBM (Additional file 1: Fig. S3d). There was a positive correlation of mRNA levels of *FAP* and *TGFB1* in the TCGA and CGGA RNAseq datasets of GBM (Additional file 1: Fig. S3e).

**Fig. 3:**
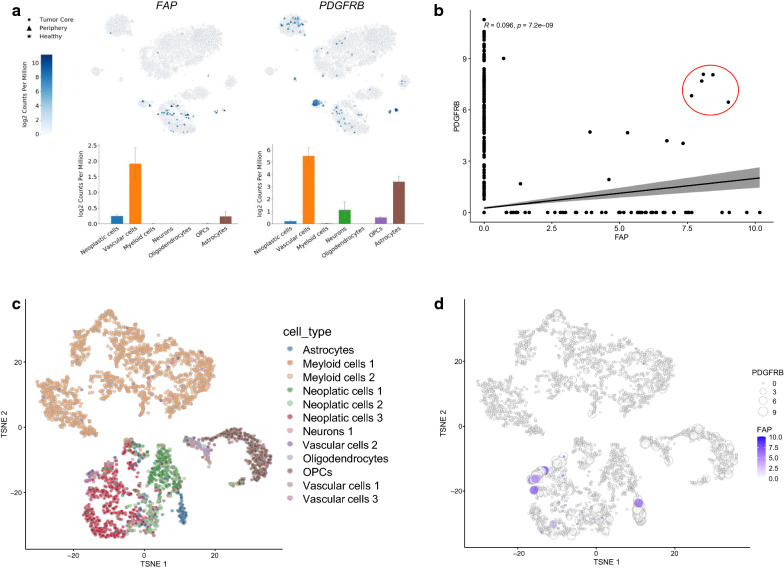
Single cell RNA sequencing analysis identifies FAP^high^/PDGFRB^high^ cells as non-endothelial vascular cells in GBM. **a** Mapping of FAP and pericyte markers onto tSNE cell clusters of single cell RNAseq analysis of GBM. Plots show RNA levels of different cell types. **b** Correlation analysis of *FAP* and *PDGFRB* transcript levels identifies a subset of cells that co-express *FAP* and *PDGFRB* at high levels (red circle). R, Pearson’s R. **c** Annotation of the 18 clusters identified by a new tSNE analysis with the 12 distinct cell types described by Darmanis et al. **d** Integration of *FAP* and *PDGFRB* expression levels into the new tSNE map to determine the phenotype of *FAP*^high^/*PDGFRB*^high^ cells

To gain further insights into the identity of the FAP +/PDGFRβ+ cells, we analyzed single cell RNAseq data and found that both FAP and PDGFRβ had the highest expression in vascular cell populations in GBM (Fig. 3a). Additional pericyte markers, CD13 (*ANPEP*) and *CD248*, also showed elevated mRNA levels in the vascular cell populations, with striking vascular selectivity observed with *CD248* (Additional file 1: Fig S4a). Interestingly, one of the commonly used CAF markers, *S100A4* (FSP1), exhibited strong expression within the myeloid population in GBM (Additional file 1: Fig S4a). Further analysis of the single cell RNA sequencing data revealed a small subset of cells (5 cells) that co-express *FAP* and *PDGFRB* at high levels (Fig. 3b). To define the identity of these *FAP*^high^/*PDGFRB*^high^ cells, we performed t-distributed stochastic neighbor embedding (tSNE) that yielded 18 distinct clusters in a 2D map of all cells (Additional file 1: Fig S4b). By overlaying the 12 phenotypically distinct clusters from the Darmanis analysis onto these clusters (Fig. 3c) and examining the expression levels of *FAP* and *PDGFRB* in individual cells, we demonstrated that 4 of the 5 *FAP*^high^/*PDGFRB*^high^ cells were mapped to Vascular cells type 3, which, together with Vascular cells type 1, constitute non-endothelial components of non-neoplastic vascular cell types (Fig. 3d) [31]. Furthermore, bulk RNAseq analysis of the TCGA and CGGA GBM datasets showed highly significant positive correlation in mRNA levels of *FAP* and *PDGFRB*, *CD248*, or CD13 (*ANPEP*) (Additional file 1: Fig S5). Together, these observations suggest that a subset of FAP+ cells present in GBM represents GBM-associated pericytes.

### FAP-positive cells are tumor-associated pericyte-like stromal cells in mouse GBM

**Fig. 4:**
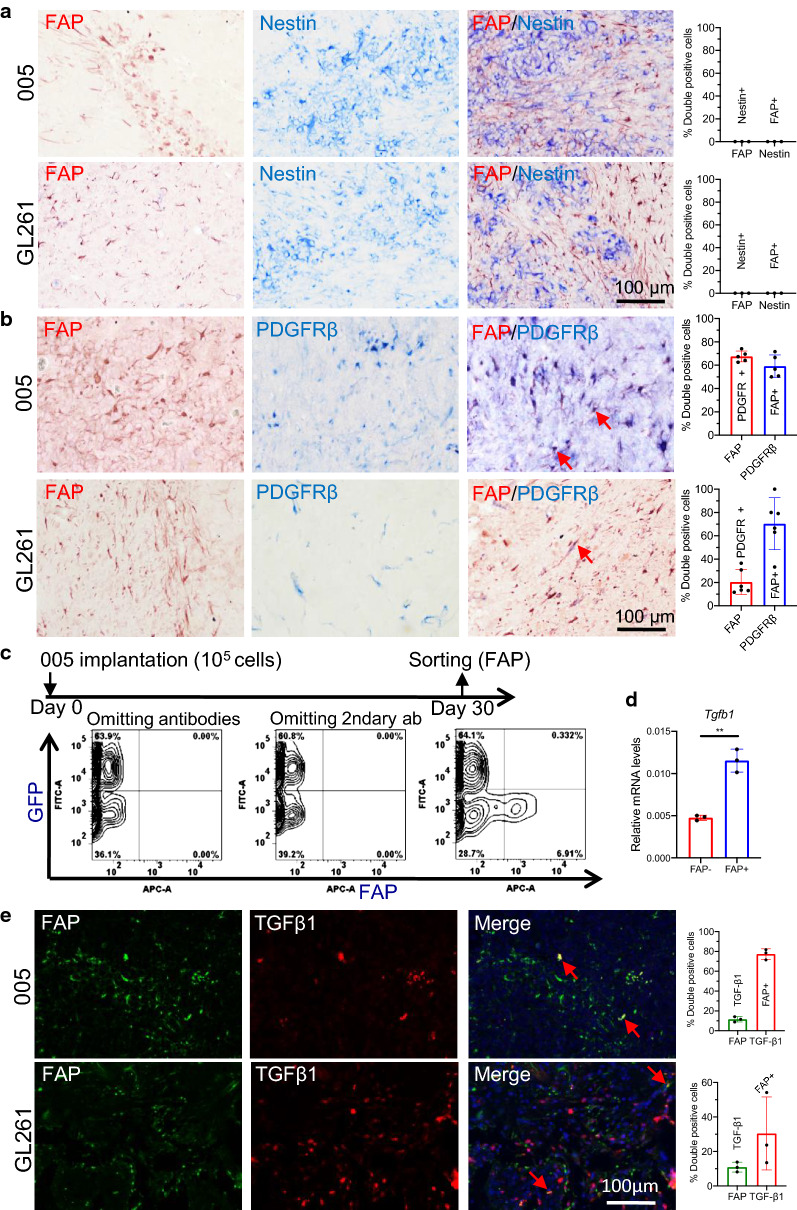
The pericyte characteristics of FAP+ cells in mouse glioblastoma. **a**, **b** Double immunohistochemistry (IHC) of FAP (red)/nestin (blue) (none were detected) (**a**) and FAP (red)/PDGFRβ (blue) (**b**) in mouse glioblastoma, 005 and GL261. Red arrows point to representative double positive cells. **c** Animal experiment scheme (top) and flow cytometry analysis (bottom) of the distribution of FAP+ and GFP+ 005 cells. **d** qRT-PCR analysis of TGF-β1 (Tgfb1) RNA levels in FAP+ and FAP- cells after sorting with flow cytometry. **p < 0.01 (*t* test). **e** Double immunofluorescence of FAP (green) and TGF-β1 (red) in mouse glioblastoma. **a**, **b**, **e** Quantification plots on the right. Error bars, SD

We next characterized FAP+ cells in mouse GBMs to determine if mouse models recapitulate the biology of FAP+ in human GBM. In 005 and GL261 GBM models in immunocompetent C57BL/6 mice, we found FAP+ cells typically featured an elongated shape and were frequently (accounting for up to 10% of all cells) distributed throughout the tumors without forming clusters (Fig. 4a). Interestingly, double IHC demonstrated no colocalization of FAP and nestin in both 005 and GL261 GBMs (Fig. 4a). In contrast, the majority (60–70%) of PDGFRβ+ cells were co-stained with FAP in both GBM models, with ~ 20% (GL261) and ~ 65% (005) of FAP+ cells being PDGFRβ+ (Fig. 4b). Further characterization revealed that murine FAP+ cells were negative for glioma/oligodendrocyte marker olig2 and GBM-associated macrophage marker Arg1 (Additional file 1: Fig S6a, b). There was very limited co-labeling of FAP and astrocyte marker GFAP (Additional file 1: Fig S6c). These findings suggest that in these murine GBMs, FAP+ cells were almost uniformly pericyte-like stromal cells, without substantive participation of neoplastic cells. Indeed, flow cytometry of acutely dissociated intracerebral tumors generated with 005 GBM cells engineered to stably express GFP showed that FAP+ populations, comprising about 6% of all living cells, were confined to GFP-negative cell subsets (Fig. 4c). Collectively our data indicate that FAP+ cells in these murine GBMs represent non-neoplastic, pericyte-like stromal cells.

Next we asked whether murine GBM-associated FAP+ cells over-expressed TGF-β1. Quantitative RT-PCR analysis of FAP+ versus FAP- cells after flow cytometric sorting of acutely dissociated 005 GBM showed that FAP+ cells expressed 2.5 times higher *Tgfb1* mRNA than FAP-cells (Fig. 4d). Double IF staining for FAP and TGF-β1 in the GBM models showed that about 10% of FAP+ cells also stained for TGFβ1, while ~ 30% (GL261) and ~ 75% (005) of TGF-β1+ cells were FAP+ (Fig. 4e). Thus, murine GBM-associated FAP+ cells comprise stromal cells with a pericytic phenotype and not neoplastic cells, some of which express TGF-β1.

### Oncolytic adenovirus can target GBM associated FAP+ cells in vitro and in vivo

**Fig. 5:**
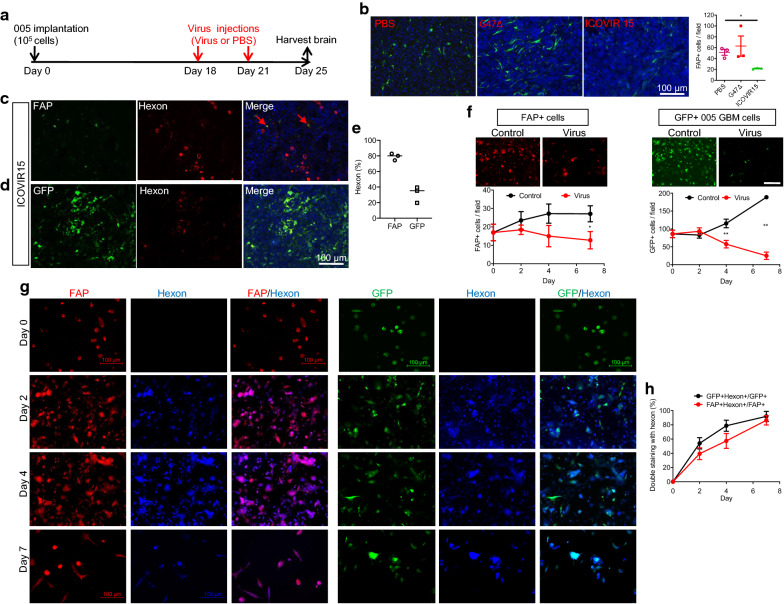
Oncolytic adenovirus targets glioblastoma-associated FAP+ cells in vitro and vivo. **a** Animal experiment schema. **b** Immunofluorescence of FAP+ in 005 murine GBM after treatment with PBS, G47∆, and ICOVIR15. Quantification of FAP+ cells on the right. *p < 0.05 (*t* test). **c**, **d** Double immunofluorescence of FAP and adenovirus marker hexon (**c**) and GFP (005 marker) and hexon (**d**) after injections of ICOVIR15. Arrows point to representative double positive cells. **e** Hexon positivity (%) in FAP+ cells and GFP+ cells. **f** Cell growth/kill curves of FAP+ cells (left) and GFP+ 005 cells (right) after treating 005 tumor-derived acutely dissociated cells with mock (control) or ICOVIR15 (virus) in vitro. Representative microscopic images on day 7 are presented on top. Scale bar: 100 μm. *p < 0.05; **p < 0.01 (*t* test). **g** Immunofluorescence of FAP (red) and Hexon (blue), and GFP (green) and hexon (blue) during the time course following in vitro infection of 005 tumor dissociated cells with ICOVIR15. Scale bar: 100 μm. **h** Change in the fraction of hexon+ cells (%) within GFP+ 005 cells and FAP+ cells post-infection with ICOVIR15 in vitro. Error bars, SD

CAFs were previously shown to be able to be targeted by several oncolytic viruses and supported enhanced viral replication compared to normal fibroblasts [22]. Thus, we tested whether DNA oncolytic viruses could target GBM-associated FAP+ stromal pericytes. We decided to test oncolytic herpes simplex virus (G47∆) and adenovirus (ICOVIR15) as we have previously shown their anti-tumor activity against mouse GBM [21, 39]. We injected G47∆ and ICOVIR15 into orthotopic 005 GBM in mice (Fig. 5a) and assessed the effects on FAP+ cells. We found that the number of FAP+ cells in the tumors decreased when treated with ICOVIR15, while there was no change in FAP+ cell number after G47∆ treatment (Fig. 5b). Double immunofluorescence for FAP and adenovirus hexon protein showed that 80% of FAP+ cells were positive for hexon, indicating that FAP+ cells were efficiently infected with ICOVOR15 and probably supported virus replication (Fig. 5c–e), while the percent of infected GFP+ tumor cells (hexon/GFP double positivity) was lower (Fig. 5d, e).

To confirm the susceptibility of GBM-associated FAP+ cells to oncolytic adenovirus, 005 GBM intracerebral tumors were excised and acutely dissociated cells derived from the tissue were subjected to in vitro culture and ICOVIR15 or mock infection. Serial immunofluorescence observations showed that ICOVIR15 inhibited the viability of both GFP+ tumor cells and FAP+ cells over time (Fig. 5f, Additional file 1: Fig S7). Dual immunofluorescence for FAP and hexon, or GFP and hexon showed that double positive FAP +/hexon+ and GFP +/hexon+ cells both increased over time till day 7, further supporting the susceptibility of both 005 cells and FAP+ cells to ICOVIR15 (Fig. 5g, h). Thus, oncolytic adenovirus ICOVIR15 exhibited the ability to target not only GBM cells, but also GBM-associated FAP+ cells in vitro and in vivo.

## Discussion

FAP is traditionally linked with tissue repair and extracellular matrix remodeling due to its dipeptidyl peptidase activity [40], however, it is one of the most upregulated genes in the tumor stroma and widely considered one of the most reliable CAF-markers [9, 41]. In this work, we show that FAP+ cells are increased in human GBM and have clinical prognostic value in glioma. Our pathological characterization of human and mouse GBM reveals that the major FAP+ populations are stromal cells co-labeled with PDGFRβ in both patient and mouse models. However, FAP+ populations within human GBM are clearly heterogenous as FAP/nestin double positive cells frequently co-exist along with FAP +/PDGFRβ+ cells, suggesting FAP expression by neoplastic cells with a mesenchymal phenotype [42]. Single cell RNA analysis showed neoplastic FAP+ cells outnumbering vascular FAP+ cells, which may have been confounded by technical difficulties of processing vascular cells for single cell preparation. GBM stem cells have been shown to have the ability to trans-differentiate into pericytes [43], which may express FAP. In *IDH1*-mutant GBM, a substantive fraction of FAP+ cells were neoplastic cells labeled with *IDH1* R132H. IHC of human GBM revealed a significant fraction of FAP+ cells showing a characteristic peri-vascular distribution, consistent with previous reports that FAP+ cells in GBM are predominantly located in perivascular areas [17, 19]. Our analysis of single cell RNA data of GBM mapped a small subset of *FAP*^high^/*PDGFRB*^high^ cells in non-neoplastic, non-endothelial vascular cell types. These results, together with the knowledge that PDGFRβ is a well-documented brain pericyte marker [33] support that FAP +/PDGFRβ+ cells observed in human GBM represent tumor-associated pericytes. In the two mouse models tested, we did not detect significant neoplastic cell populations expressing FAP. In both mouse GBM models, FAP+ cells displayed non-clustered, characteristic elongated cell morphology, negativity for astrocyte (GFAP) and macrophage (Arg1) markers, and had infrequent Ki67 co-labeling indicative of low proliferative activity. PDGFRβ was used to mark tumor-associated pericytes in GL261 GBM [44]. Together, our findings and others suggest that FAP +/PDGFRβ+ cells in mouse GBM are likely to be pericytes as their human counterparts, which, however, requires validation by future research. We also show that the expression of FAP and αSMA do not co-localize. αSMA is another commonly used marker for CAFs as well as brain perivascular cells, and is linked to a myofibroblast phenotype [9, 33]. Our observation that FAP+ and αSMA+ cells are distinct in GBM is in accord with our knowledge that CAFs in cancer are highly heterogenous in marker status and function [4, 9, 45], and suggests that mesenchymal stromal cells in the GBM tumor microenvironment are also heterogenous.

Constituting a component of brain micro-vessels, brain pericytes have been shown to have versatile functions, including the maintenance of the blood–brain barrier and the regulation of immunity and inflammation in the CNS [46, 47]. Emerging research supports the role pericytes in the GBM microenvironment play to promote tumor growth and regulate drug penetration [43, 48, 49]. Recent studies further showed that brain tumor cells induced pericytes to secrete high levels of immunosuppressive cytokines, such as IL-10 and TGFβ, suggesting that intimate cross-talk between GBM cells and pericytes reprogram GBM-associated pericytes to acquire immunosuppressive properties [49–51]. These reports are consistent with our finding that FAP+ cells, despite accounting for at most only 10% of total cells, participate in the production of TGFβ in GBM. In this regard, the contribution of FAP+ stromal cells to GBM evasion of host anti-tumor immunity appears analogous to the well-documented roles of CAFs in enhancing immuno-suppression in solid cancers [5, 6, 35].

Oncolytic virus (OV) immunotherapy is an effective strategy for cancer, which uses several mechanisms of therapeutic action, including direct selective killing of cancer cells as well as elicitation of anti-tumor immune responses [23, 52]. Currently, oHSV T-VEC is FDA-approved for advanced melanoma [53]. Several other recombinant OVs are in clinical trials for cancers including GBM, and some such as oncolytic adenovirus DNX-2401 are beginning to demonstrate safety and potential clinical benefits for patients [54, 55]. Herein we demonstrate that the oncolytic adenovirus ICOVIR15 that is similar to DNX-2401 can infect and reduce GBM-associated FAP+ cells in the 005 mouse GBM model in vivo. Using freshly isolated FAP+ cells from mouse GBM, we verified the susceptibility of FAP+ stromal cells to ICOVIR15 ex vivo. Due to high-level, cell surface expression of FAP in cancer-promoting CAFs, FAP has been considered a prime therapeutic target in the cancer stroma with potential for clinical application [56, 57]. Strategies to target FAP+ CAFs include small molecule FAP inhibitors, monoclonal antibodies against FAP, and other immunotherapies using FAP DNA vaccines, FAP-directed CAR-T cells or bi-specific T cell engagers, with some approaches having been evaluated in clinical trials [45, 57]. Unlike these targeted modalities, OV is unique as its primary targets are usually considered to be neoplastic cells. However, OV targets can be extended to cancer stromal cells since cytokine-mediated reciprocal cross-talk between cancer cells and CAFs allowed CAFs to become permissive to OVs and rendered cancer cells more permissive [22]. Our work here is the first to demonstrate the ability of OV to kill tumor-associated stromal cells in GBM. While replication of ΔE1A oncolytic adenovirus depends on aberrant Rb-E2F signaling, activation of E2F transcription factors is not cancer specific as the role of E2F extends beyond cell cycle progression [58]. Our findings warrant further research to validate this new mechanism of action of oncolytic adenovirus in patient specimens from clinical trials. Whether viral targeting of GBM stroma contributes to improved therapeutic efficacy is a key question that will need to be addressed.

In conclusion, our work identified FAP/PDGFRβ dual positive tumor-associated pericytes as a distinct stromal cell type in the GBM tumor microenvironment. Oncolytic adenovirus can target these GBM associated FAP+ cells, and the demonstration of such ability could provide translational insight into improving the treatment of GBM.

## Supplementary information

**Additional file 1.** **Supplementary Figure S1. The presence of FAP-positive cells in human glioma tissue** **a**, FAP mRNA levels of IDH wild type and mutant gliomas in the TCGA and CGGA datasets. Analysis at GlioVis. ***p < 0.001. **b**, Immunohistochemistry (IHC) of FAP in human glioma tissues from 12 additional patients. **Supplementary Figure S2. Characterization of FAP+ cells in human glioblastoma.** **a**, Double immunofluorescence of FAP (green) and nestin (red), showing double positive cells in MGG7 GBM. Arrows, double-positive cells. Quantification on the bottom. **b**-**d**, Double immunofluorescence of FAP and astrocytes marker GFAP (**b**), tumor proliferation maker Ki67 (**c**), and perivascular marker α-SMA (**d**). Red arrows point to representative double positive cells. Quantification on the right. (no FAP+/α-SMA+ cells or FAP+/GFAP+ MGG90 cells). Error bars, SD. **Supplementary Figure S3. Characterization of FAP+ cells in human glioblastoma.** **a**-**c**, Double immunohistochemistry of FAP (red or brown) and IDH1R132H (blue) (**a**), nestin (blue) (**b**), and PDGFRβ (blue) (**c**) in MGG81 (*IDH*-mutant). Red arrows point to representative double positive cells. Quantification on the right. **d**, Double immunofluorescence of FAP (red) and TGF-β1 (green) and quantification of positivity in MGG153 (*IDH*-wild-type). Error bars, SD. **e**, Correlation between FAP and TGFB1 mRNA levels in TCGA and CGGA datasets of GBM (RNAseq). Analysis at Gliovis. R, Pearson’s R. **Supplementary Figure S4. Single cell RNA sequencing analysis of human glioblastoma (Darmanis et al data).** **a**, Mapping of pericyte markers CD13 (ANPEP) and CD248 and CAF maker FSP (*S100A4*) onto tSNE cell clusters of single cell RNAseq analysis of GBM. **b**, New 2D tSNE clustering of all cells, generating 18 clusters of cells. **Supplementary Figure S5. Correlation between FAP and pericyte makers in bulk RNA datasets.** Correlation between *FAP* and *PDGFRB*, *CD248*, and *ANPEP* in the TCGA and CGGA GBM datasets is shown. Analysis at Gliovis. R, Pearson’s R. **Supplementary Figure S6. Biological characteristics of FAP+ cells in mouse glioblastoma.** **a**-**c**, Double immunofluorescene of FAP with oligodendrocyte/glioma marker olig2 (**a**), M2 macrophage marker Arg1 (**b**), and astrocyte marker GFAP (**c**). Quantification plots on the right. (no FAP+/Olig2+ or FAP+/Arg1+ cells). Error bars, SD. **Supplementary Figure S7. Oncolytic adenovirus targets mouse FAP+ cells and glioblastoma cells in vitro.** Immunofluorescence for FAP and GFP at different time-point after ICOVIR15 treatment of 005 GBM-derived cells in vitro. See Figure 5f for quantification of cell number.

## Data Availability

Data sharing is not applicable to this article, as no datasets were generated during the current study.
