# Supplementary material for: Characterization and oncolytic virus targeting of FAP-expressing tumor-associated pericytes in glioblastoma
Source: Acta Neuropathol Commun. 2020 Dec 11;8:221. doi: 10.1186/s40478-020-01096-0 (PMC7730751; doi:10.1186/s40478-020-01096-0)
Supplement: Supplementary file 1 — Additional file 1. Supplementary Figure S1. The presence of FAP-positive cells in human glioma tissue a, FAP mRNA levels of IDH wild type and mutant gliomas in the TCGA and CGGA datasets. Analysis at GlioVis. ***p < 0.001. b, Immunohistochemistry (IHC) of FAP in human glioma tissues from 12 additional patients. Supplementary Figure S2. Characterization of FAP+ cells in human glioblastoma. a, Double immunofluorescence of FAP (green) and nestin (red), showing double positive cells in MGG7 GBM. Arrows, double-positive cells. Quantification on the bottom. b-d, Double immunofluorescence of FAP and astrocytes marker GFAP (b), tumor proliferation maker Ki67 (c), and perivascular marker α-SMA (d). Red arrows point to representative double positive cells. Quantification on the right. (no FAP+/α-SMA+ cells or FAP+/GFAP+ MGG90 cells). Error bars, SD. Supplementary Figure S3. Characterization of FAP+ cells in human glioblastoma. a-c, Double immunohistochemistry of FAP (red or brown) and IDH1R132H (blue) (a), nestin (blue) (b), and PDGFRβ (blue) (c) in MGG81 (IDH-mutant). Red arrows point to representative double positive cells. Quantification on the right. d, Double immunofluorescence of FAP (red) and TGF-β1 (green) and quantification of positivity in MGG153 (IDH-wild-type). Error bars, SD. e, Correlation between FAP and TGFB1 mRNA levels in TCGA and CGGA datasets of GBM (RNAseq). Analysis at Gliovis. R, Pearson’s R. Supplementary Figure S4. Single cell RNA sequencing analysis of human glioblastoma (Darmanis et al data). a, Mapping of pericyte markers CD13 (ANPEP) and CD248 and CAF maker FSP (S100A4) onto tSNE cell clusters of single cell RNAseq analysis of GBM. b, New 2D tSNE clustering of all cells, generating 18 clusters of cells. Supplementary Figure S5. Correlation between FAP and pericyte makers in bulk RNA datasets. Correlation between FAP and PDGFRB, CD248, and ANPEP in the TCGA and CGGA GBM datasets is shown. Analysis at Gliovis. R, Pearson’s R. Supplementary Figure S6 [file 40478_2020_1096_MOESM1_ESM.pdf]

Supplementary Figure S1

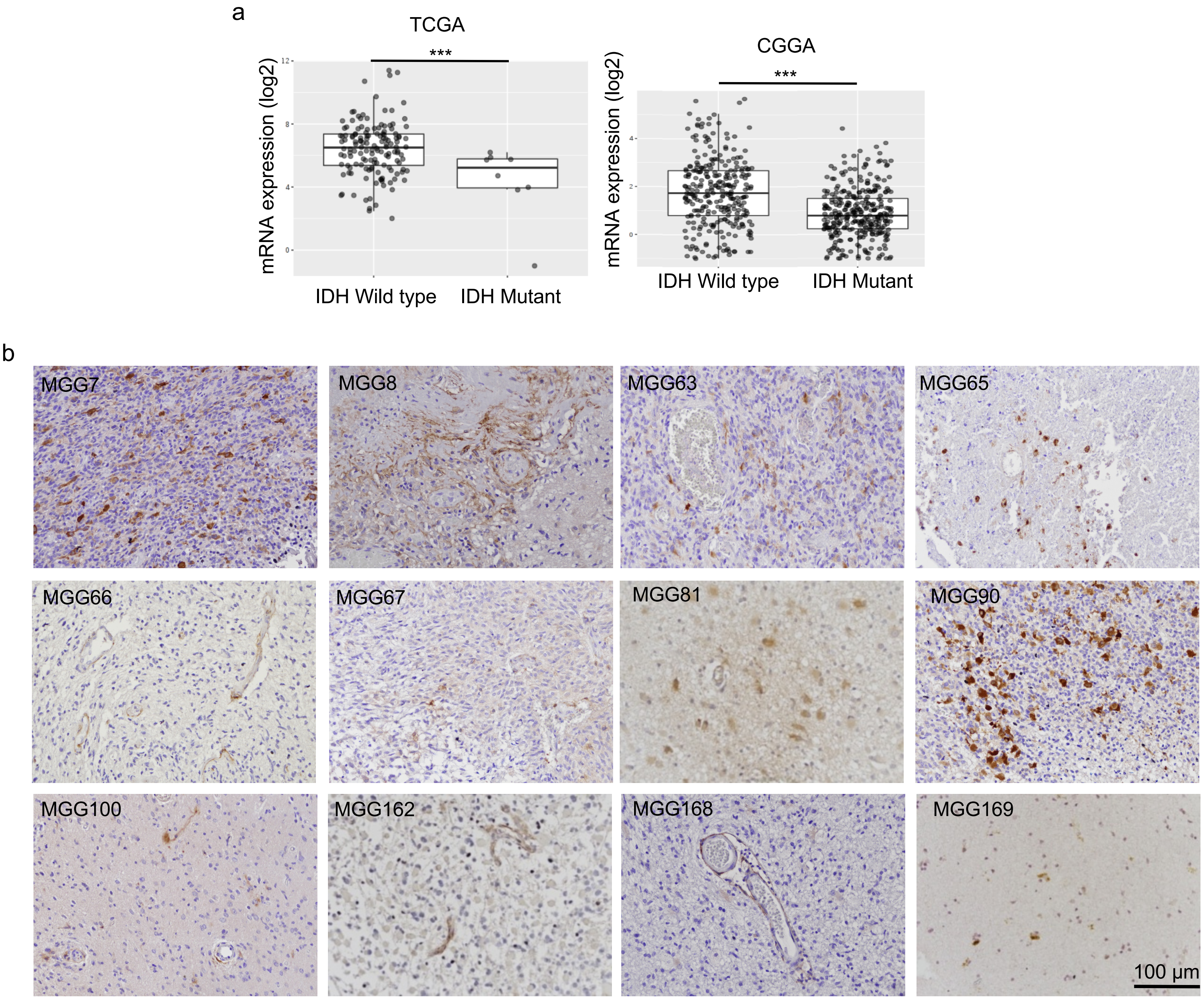

Supplementary Figure S1. The presence of FAP-positive cells in human glioma tissue. a, FAP mRNA levels of IDH wild type and mutant gliomas in the TCGA and CGGA datasets. Analysis at GlioVis. ***p<0.001. **b**, Immunohistochemistry (IHC) of FAP in human glioma tissues from 12 additional patients.

Supplementary Figure S2

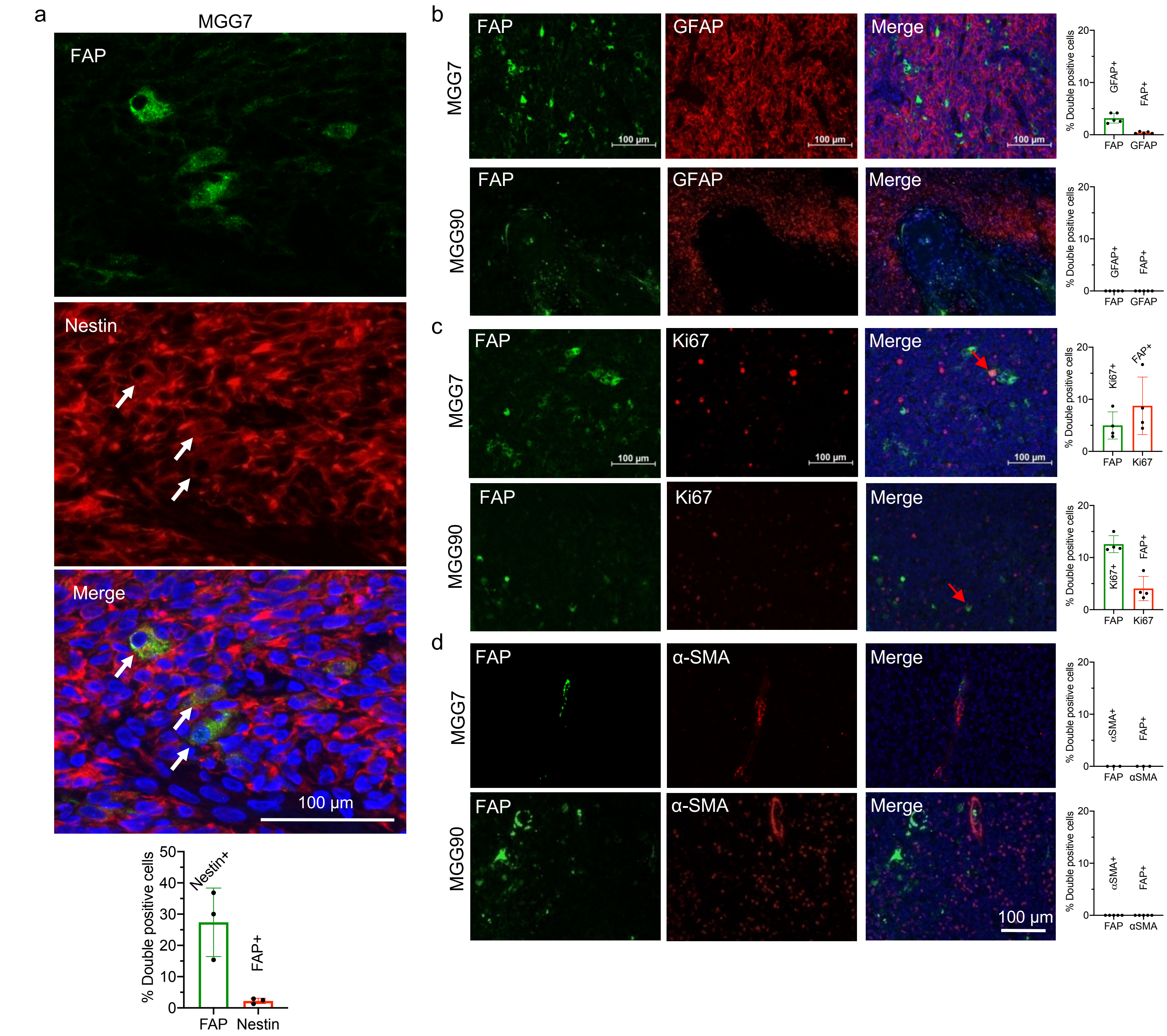

Supplementary Figure S2. Characterization of FAP+ cells in human glioblastoma. a, Double immunofluorescence of FAP (green) and nestin (red), showing double positive cells in MGG7 GBM. Arrows, double-positive cells. Quantification on the bottom. b-d, Double immunofluorescence of FAP and astrocytes marker GFAP (b), tumor proliferation maker Ki67(c), and perivascular marker α-SMA (d). Red arrows point to representative double positive cells. Quantification on the right. (no FAP+/α-SMA+ cells or FAP+/GFAP+ MGG90 cells). Error bars, SD.

Supplementary Figure S3

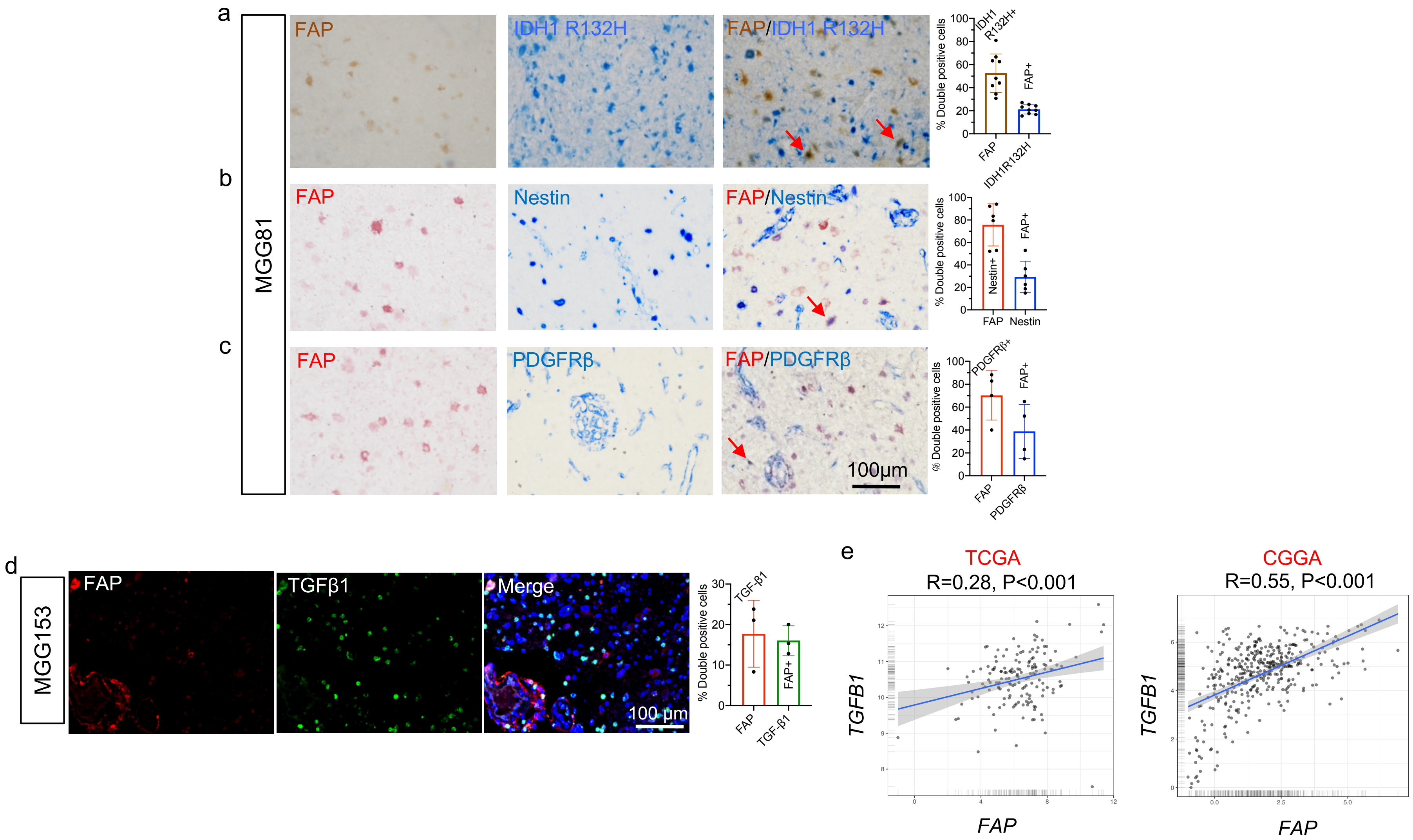

Supplementary Figure S3. Characterization of FAP+ cells in human glioblastoma.
a-c, Double immunohistochemistry of FAP (red or brown) and IDH1R132H (blue)(a), nestin (blue)(b), and PDGFRβ (blue)(c) in MGG81 (*IDH1*-mutant). Red arrows point to representative double positive cells. Quantification on the right. d, Double immunofluorescence of FAP (red) and TGF-β1 (green) and quantification of positivity in MGG153 (*IDH*-wild-type). Error bars, SD. e, Correlation between FAP and TGFB1 mRNA levels in TCGA and CGGA datasets of GBM (RNAseq). Analysis at Gliovis. R, Pearson's R.

Supplementary Figure S4

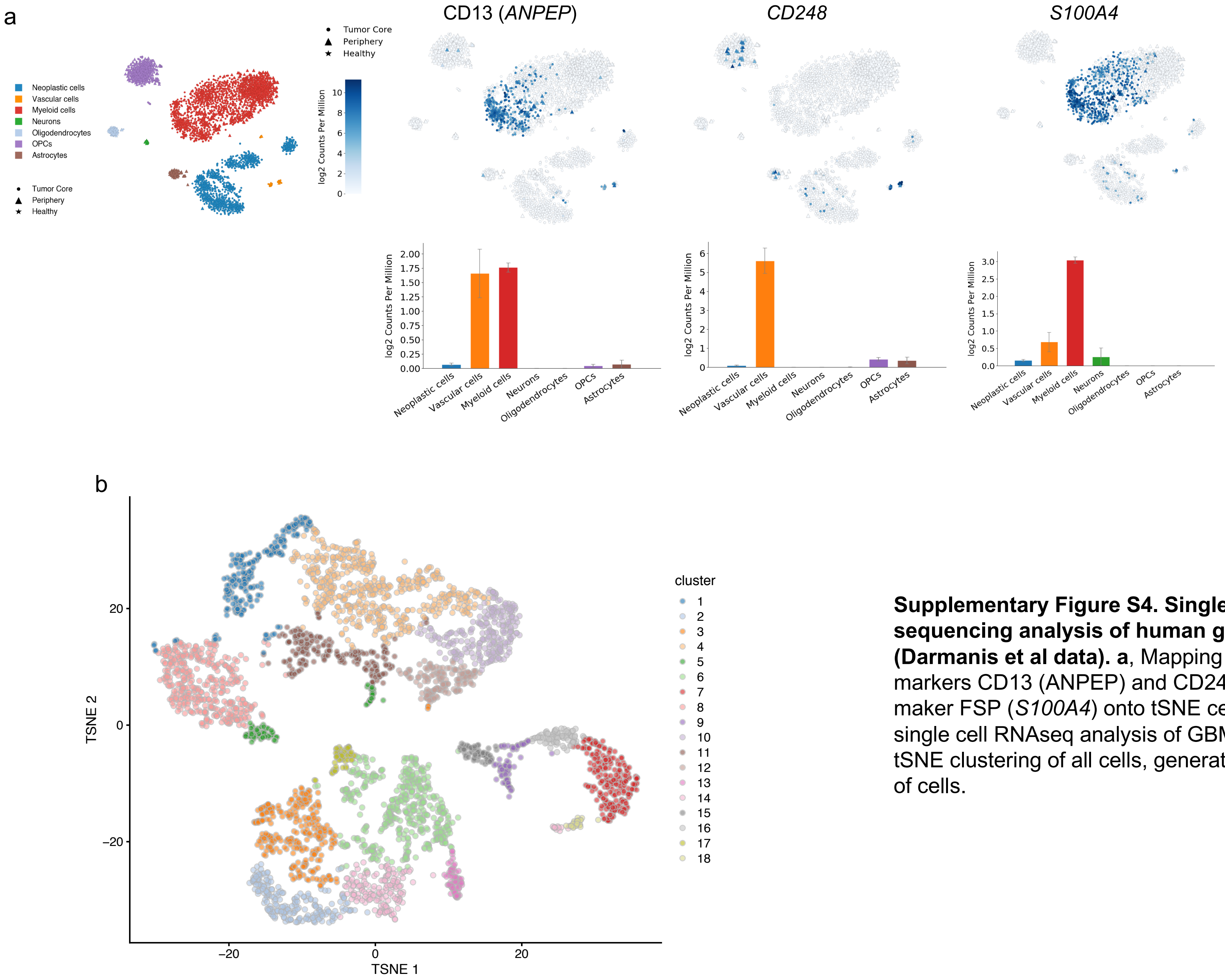

Supplementary Figure S4. Single cell RNA sequencing analysis of human glioblastoma (Darmanis et al data). a, Mapping of pericyte markers CD13 (ANPEP) and CD248 and CAF maker FSP (*S100A4*) onto tSNE cell clusters of single cell RNAseq analysis of GBM. **b**, New 2D tSNE clustering of all cells, generating 18 clusters of cells.

Supplementary Figure S5

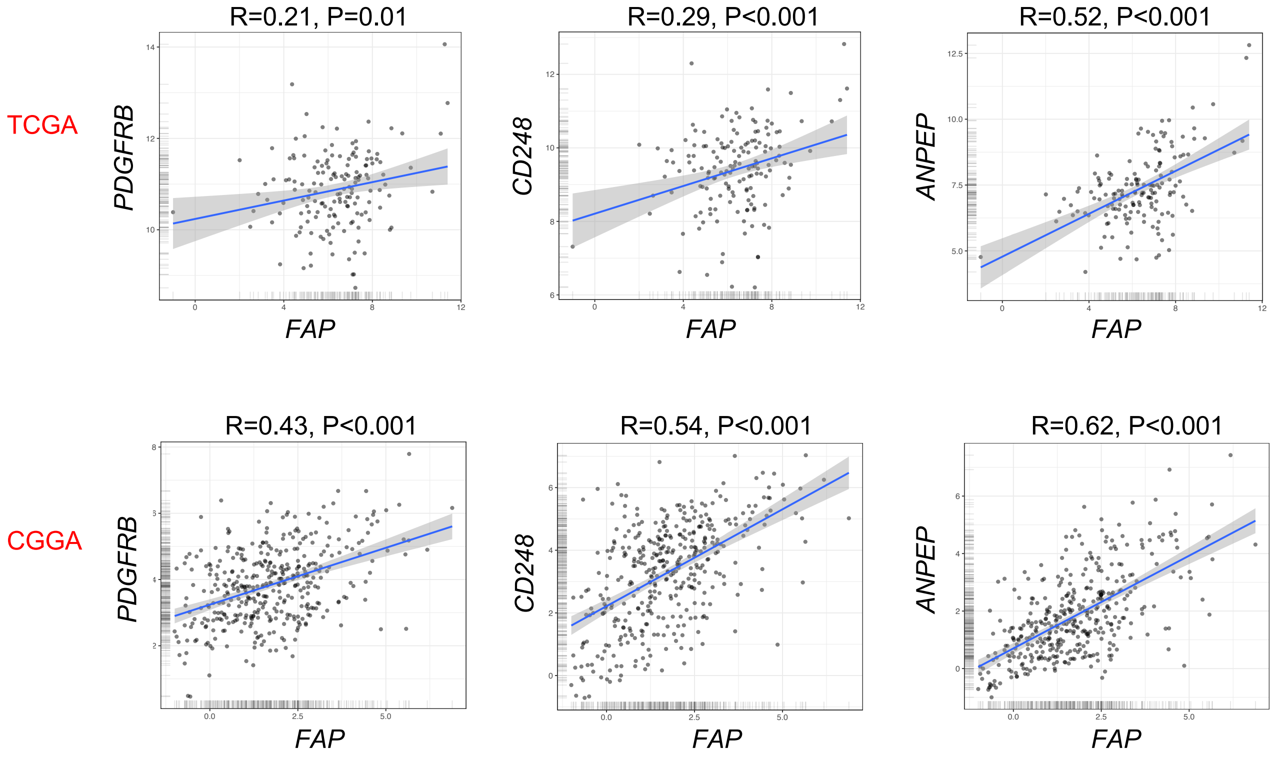

Supplementary Figure S5. Correlation between FAP and pericyte makers in bulk RNA datasets. Correlation between *FAP* and *PDGFRB*, *CD248*, and *ANPEP* in the TCGA and CGGA GBM datasets is shown. Analysis at Gliovis. R, Pearson's R.

Supplementary Figure S6

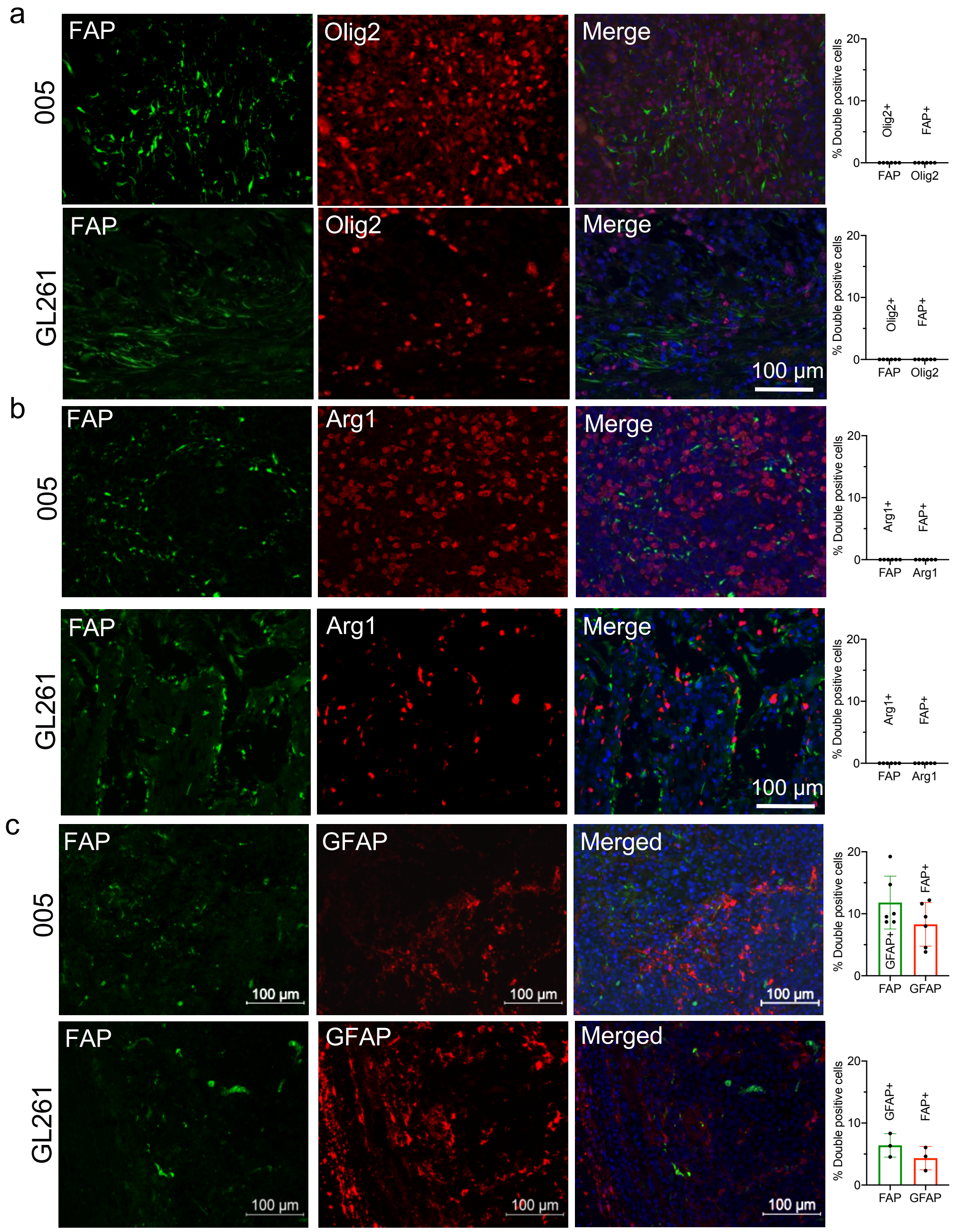

Supplementary Figure S6. Biological characteristics of FAP+ cells in mouse glioblastoma.
a-c, Double immunofluorescene of FAP with oligodendrocyte/glioma marker olig2 (a), M2 macrophage marker Arg1 (b), and astrocyte marker GFAP (c). Quantification plots on the right. (no FAP+/Olig2+ or FAP+/Arg1+ cells). Error bars, SD.

Supplementary Figure S7

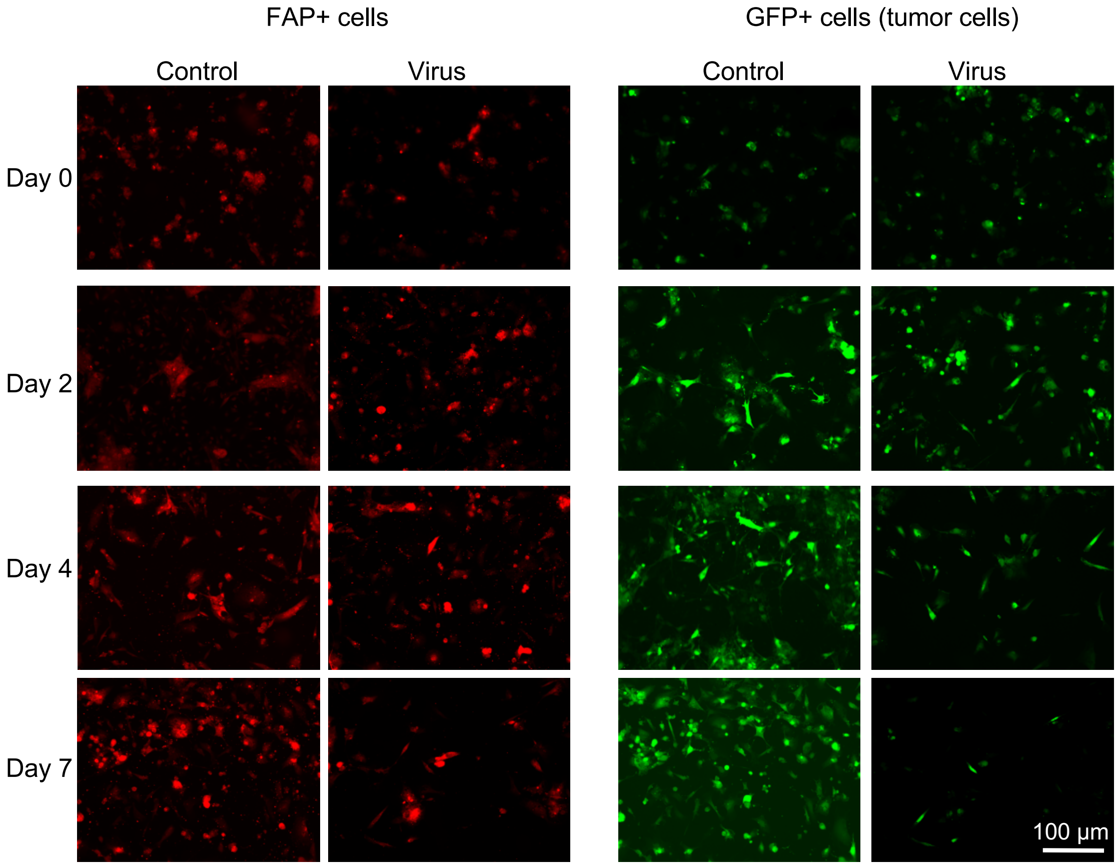

Supplementary Figure S7. Oncolytic adenovirus targets mouse FAP+ cells and glioblastoma cells in vitro. Immunofluorescence for FAP and GFP at different time-point after ICOVIR15 treatment of 005 GBM-derived cells in vitro. See Figure 5f for quantification of cell number.

Supplementary Table S1

Primary antibodies used in this work

Antibody	Source	Catalogue number	Host species	Note
FAP	Abcam	Ab207178	Rabbit	For human FAP
FAP	Aviva systems bio	OAAF01790	Rabbit	For mouse FAP
Nestin	Santa Cruz	SC23927	Mouse	For human nestin
Nestin	Millipore Sigma	MAM353	Mouse	For mouse nestin
Ki67	Dako	65742	Mouse	
αSMA	Thermo Fisher	14-9760-82	Mouse	
IDH1 R132H	ScyTek	DIA-H09	Mouse	
PDGFRβ	Cell Signaling	3169	Rabbit	
TGFβ1	R&D Systems	MAB240	Mouse	
GFAP	Sigma	G3893	Mouse	
GFP	Abcam	Ab183734	Rabbit	
Arg1	Santa Cruz	Sc-271430	Mouse	
Olig2	Millipore	MABN50	Mouse	
Hexon	Millipore Sigma	AB1056	Goat	
